# Associations of Mental Health and Chronic Physical Illness During Childhood With Major Depression in Later Life

**DOI:** 10.1093/geroni/igab046.105

**Published:** 2021-12-17

**Authors:** Rachel Bergmans, Jacqui Smith

**Affiliations:** University of Michigan, University of Michigan, Michigan, United States

## Abstract

While poor health in childhood has implications for mental health years later, less is known regarding its long-term impact. We determined whether childhood chronic physical illness burden was associated with major depression (MD) in later life (i.e., >50 years), and tested mediation by childhood mental health status using path analysis. Data came from the 2016 U.S. Health and Retirement Study (n=18,047). One standard deviation increase in childhood chronic physical illness burden was associated with 1.21 (95% CI = 1.12, 1.30) times higher odds of MD in later life. Childhood mental health status explained 57.8% (95% CI: 35.2, 80.4) of this association. Results indicated that the relationship of chronic physical illness burden in childhood with MD in later life was mediated by childhood mental health status. Whether greater screening for psychiatric-related symptoms in childhood or review of health histories in later life can reduce the burden of MD requires further study.

